# Association of the tandem polymorphisms (rs148314165, rs200820567) in *TNFAIP3* with chronic hepatitis B virus infection in Chinese Han population

**DOI:** 10.1186/s12985-017-0814-5

**Published:** 2017-08-07

**Authors:** Na Li, Ying Shi, Pingping Zhang, Jiao Sang, Fang Li, Huan Deng, Yi Lv, Qunying Han, Zhengwen Liu

**Affiliations:** 1 Department of Infectious Diseases, First Affiliated Hospital of Xi’an Jiaotong University, 277 Yanta West Road, Xi’ an, Shaanxi Province 710061 China; 2 Maternal and Children Health Hospital of Tongchuan, Tongchuan, Shaanxi 727000 China; 3 Department of Hepatobiliary Surgery, First Affiliated Hospital of Xi’an Jiaotong University, Xi’an, Shaanxi 710061 China; 4 Institute of Advanced Surgical Technology and Engineering, Xi’an Jiaotong University, Xi’ an, Shaanxi 710061 China

**Keywords:** Hepatitis B virus, *TNFAIP3*, Polymorphism, Susceptibility, Clinical disease

## Abstract

**Background:**

Chronic hepatitis B virus (HBV) infection remains an important public health issue. A20, a ubiquitin-editing protein encoded by tumor necrosis factor alpha-inducible protein 3 (*TNFAIP3*) gene, is complicated in HBV infection and liver injury. The tandem polymorphisms (rs148314165, rs200820567), deletion T followed by a T to A transversion and collectively referred to as TT > A in *TNFAIP3*, may attenuate A20 expression.

**Methods:**

The rs148314165 and rs200820567 polymorphisms were examined using PCR amplification followed by direct sequencing in 419 patients with chronic HBV infection, 77 HBV infection resolvers and 175 healthy controls of Chinese Han ethnicity.

**Results:**

The genotypes and alleles of rs148314165 and rs200820567 polymorphisms determined and the haplotypes constructed were consistently identical, confirming the reliable determination of the TT > A variant. The genotypes of rs148314165 and rs200820567 in HBV patients, HBV infection resolvers and healthy controls are in Hardy-Weinberg equilibrium (*P* > 0. 05). The patients with chronic HBV infection had higher frequency of TT > A variant than healthy controls (6.6% vs. 3.4%; OR, 1.979; 95% CI, 1.046–3.742; *P* = 0.033). The frequency of TT > A variant between patients with chronic hepatitis, liver cirrhosis, and hepatocellular carcinoma had no significant differences.

**Conclusions:**

The TT > A variant of *TNFAIP3* may be associated with the susceptibility of chronic HBV infection but not the clinical diseases. Studies in large sample size of HBV patient and control populations are required to further clarify the role of this important variant in chronic HBV infection and the disease progression related to the infection.

**Electronic supplementary material:**

The online version of this article (doi:10.1186/s12985-017-0814-5) contains supplementary material, which is available to authorized users.

## Background

Infection with hepatitis B virus (HBV) may lead to acute and chronic liver diseases including liver cirrhosis and hepatocellular carcinoma (HCC) and remains an important public health issue. [1]. The resolution or persistence of HBV infection and the development or progression of liver diseases related to the infection are considered to be associated with the host immune responses [2, 3].

A20, a ubiquitin-editing protein encoded by tumor necrosis factor (TNF) alpha-inducible protein 3 (*TNFAIP3*) gene, is critical for regulating nuclear factor kappa-B (NF-κB) activity and TNF-mediated programmed cell death [4–6]. NF-kB family members play crucial roles in the regulation of genes involved in diverse biological processes, including inflammation, immune responses, carcinogenesis and apoptosis [7], and are also complicated in HBV infection [8, 9] and HBV-related liver injury [10]. Alterations of A20 may affect the progress of HBV infection and liver diseases. For instance, HBV X protein (HBx) was indicated to be able to affect the expression of A20 and promote TNF-related apoptosis-inducing ligand (TRAIL)-induced hepatocyte apoptosis [11]. A20 mRNA expression in peripheral blood mononuclear cells was shown to be associated with progression of chronic HBV infection [12], and increased A20 expression on monocytes was shown to be associated with the severity of acute-on-chronic liver failure related to HBV infection [13]. A20 also plays an important role in normal liver tissue homeostasis. In a mouse line lacking A20 specifically in liver parenchymal cells, A20 deficiency was suggested to be associated with the development of chronic liver inflammation and enhanced hepatocyte apoptosis, and the increased susceptibility to chemically or high fat-diet-induced HCC development, establishing A20 as a crucial hepatoprotective factor [14]. A20 was also shown to promote liver regeneration [15] and suppress HCC proliferation and metastasis [16, 17].

Genetically, decreased A20 expression, as in A20 heterozygous knockout mice, causes worse outcome following two-third partial hepatectomy, ascertaining a primordial role of A20 in prompting liver regeneration [18]. Polymorphisms in the human *TNFAIP3* gene may potentially alter *TNFAIP3* expression and activity. Taking into account the role of A20 in HBV infection and liver injury, it is plausible to hypothesize that *TNFAIP3* polymorphisms may be relevant to HBV infection and HBV-related liver diseases. The *TNFAIP3* polymorphism rs2230926, a nonsynonymous common coding single-nucleotide polymorphism (SNP) in exon 3 of *TNFAIP3*, has been shown to possibly influence the mRNA expression of *TNFAIP3* and the function of TNFAIP3 and to be associated with autoimmune disorders such as systemic lupus erythematosus [19, 20]. However, investigation of *TNFAIP3* rs2230926 polymorphism in patients with chronic HBV infection did not show any association with the susceptibility of chronic HBV infection or the progression of HBV-related liver diseases [21].

Noteworthily, the tandem polymorphisms (rs148314165, rs200820567), a pair of tandem polymorphic dinucleotides, 42 kb downstream of the promoter of *TNFAIP3*, was demonstrated to result in reduced *TNFAIP3* mRNA and A20 protein expression through interfering with the delivery of NF-kB to the *TNFAIP3* promoter [22, 23]. However, whether these tandem polymorphisms (rs148314165, rs200820567) of *TNFAIP3* may affect the susceptibility of chronic HBV infection and HBV-related liver diseases remain unknown. Therefore, this study examined the possible association between the tandem polymorphisms (rs148314165, rs200820567) of *TNFAIP3* and chronic HBV infection in Chinese Han population.

## Methods

### Study subjects

Patients with chronic HBV infection included in this study was recruited from the First Affiliated Hospital of Xi’an Jiaotong University. Patients who had a history of chronic HBV infection for more than 6 months and had not been treated with nucleos(t)ide analogues or interferon (IFN)-α and other immunotherapy were eligible for inclusion. The diagnosis of the clinical diseases, namely, chronic hepatitis, liver cirrhosis, and HCC, in the patients was based on a history of HBV infection more than 6 months, and serostatus of HBV infection markers, HBV DNA level, biochemical liver function, α-fetoprotein (AFP) level, ultrasonography and/or computerized tomography (CT)/magnetic resonance imaging (MRI) and/or the pathological findings of liver biopsy. Infections with hepatitis A virus (HAV), hepatitis C virus (HCV), hepatitis E virus (HEV) and human immunodeficiency virus (HIV) were excluded in all the patients. Coexistence of autoimmune, alcoholic, drug-induced or metabolic liver disease was also excluded in all the patients. HBV infection resolvers and healthy individuals were also recruited as controls. The HBV infection resolvers were recruited from individuals who performed their routine physical examination. Healthy control individuals were recruited from voluntary blood donors. HBV infection resolvers were those who had normal liver biochemical function, seropositive results of anti-HBs and anti-HBc, and no other diseases. The healthy controls were those who had normal liver biochemical function, no histoty of hepatitis B, and no other diseases. Totally, 419 patients with chronic HBV infection, 77 HBV infection resolvers and 175 healthy controls were recruited as study subjects. All the subjects were of Chinese Han ethnicity.

### Determination of serum HBV markers, liver biochemistry and AFP levels

Serum HBV markers including HBsAg, anti-HBs, HBeAg, anti-HBe and anti-HBc, biochemical liver function including alanine transaminase (ALT) and aspartate aminotransferase (AST) levels (IU/L), serum HBV DNA levels (IU/ml), and serum AFP levels (ng/ml) were routinely determined as described previously [21].

### Genotyping of the *TNFAIP3* tandem polymorphisms (rs148314165, rs200820567)

Genomic DNA was extracted from EDTA-treated peripheral blood using TIANamp Genomic DNA Kit (Tiangen Biotech (Beijing) Co., Ltd., Beijing, China) according to manufacturer’s instruction. Genotyping of TNFAIP3 rs148314165, rs200820567 polymorphisms was carried out by DNA amplifications with polymerase chain reaction (PCR) and specific primers, followed by direct sequencing. The primers used for amplification of rs148314165 polymorphic sequence are: forward 5′ TTCTGGGTACTTCTCAACAAGATG 3′ and reverse 5′ TCCATGAGGGTATCAGGACTTC 3′, and the primers used for amplification of rs200820567 polymorphic sequence are forward 5′ ATTCCTTCATGTATGTATTGCTTTG 3′ and reverse 5′ AGGACTTCCCAAAAGGGTAAG 3′.

The PCR was carried out in a volume of 50 μl reaction, containing 1 μl template, 1 μl of each primer, 1 μl dNTP (10 mM), 5 μl Taq Buffer, 5 μl 25 mM MgCl_2_, 0.5 μl P*fu* DNA Polymerase (5 U/μl, Sangon Biotech (Shanghai) Co., Ltd. Shanghai, China), and 35.5 μl sterile double distilled water. The reaction mixture was pre-heated at 95 °C for 3 min, then amplified 35 cycles using the following program: heated at 94 °C for 30 s, annealing at 55 °C for 35 s and extension at 72 °C for 40 s, and lastly extended at 72 °C for 5 min. The amplified PCR products were purified using PCR Product Purification Kit (Sangon Biotech (Shanghai) Co., Ltd. Shanghai, China) according to the instruction of the manufacturer and then applied for sequencing. The sequences were analyzed using SeqMan software (version 5.51; DNASTAR, Inc., Madison, WI).

### Statistical analysis

Statistical analysis was performed by SPSS software version 16.0 (SPSS, Inc., Chicago, IL). The frequency of genotypes and alleles was estimated by direct gene counting method. Hardy-Weinberg equilibrium of the polymorphism was tested by χ^2^ test. Haplotypes were estimated by the SHEsis method [24, 25]. The association between polymorphisms and clinical diseases was tested using χ^2^ test for contingency tables or Fisher’s exact test where applicable. Odds ratios (OR) and its 95% confidence interval (CI) were calculated to estimate the risk conferred by a particular genotype, allele or haplotype. Association of the polymorphisms with disease phenotypes was also performed by logistic regression analysis with adjusting for covariates. A *P* value <0.05 was considered statistically significant.

## Results

### Demographics and hardy-Weinberg equilibrium of the genotypes of rs148314165, rs200820567 in the study subjects

The gender and age between patients with chronic HBV infection, HBV infection resolvers and healthy controls had no significant differences (Table 1). The genotypes of rs148314165, rs200820567 in patients with chronic HBV infection, HBV infection resolvers and healthy controls are in Hardy-Weinberg equilibrium of (*P* > 0. 05, Table 1).

**Table 1:** Demographics and Hardy-Weinberg equilibrium of the genotypes of rs148314165 and rs200820567 in patients with chronic HBV infection, HBV infection resolvers and healthy controls

	Patients (*n* = 419)	Resolvers (*n* = 77)	Controls (*n* = 175)	*P*
Sex (male/female)	312/107	53/24	123/52	0.417
Age (year, mean ± SD (range))	43.19 ± 3.77 (18–77)	44.97 ± 5.64 (19–78)	44.06 ± 5.49 (19–76)	0.362
HWE (observed vs. expected)				
rs148314165				
*P* value	0.147	0.722	0.638	
rs200820567				
*P* value	0.147	0.722	0.638	

*HBV* hepatitis B virus, *HWE* Hardy-Weinberg equilibrium

### Genotype and allele frequencies of rs148314165, rs200820567 polymorphisms in the study subjects

The genotype and allele frequencies of rs148314165, rs200820567 polymorphisms in patients with chronic HBV infection, HBV infection resolvers and healthy controls are shown in Table 2. There were no significant differences in the genotype and allele frequencies of rs148314165, rs200820567 polymorphisms between patients with chronic HBV infection and HBV infection resolvers and between HBV infection resolvers and healthy controls (Table 2). However, patients with chronic HBV infection had higher genotype Tdel of rs148314165 (13.1% vs. 6.9%, OR, 2.069, 95% CI, 1.079–3.969, *P* = 0.028) and higher allele del (6.6% vs. 3.4%; OR, 1.979; 95% CI, 1.046–3.742; *P* = 0.033) than healthy controls (Table 2). Similarly, patients with chronic HBV infection had higher genotype TA of rs200820567 (13.1% vs. 6.9%, OR, 2.069, 95% CI, 1.079–3.969, *P* = 0.028) and higher allele A (6.6% vs. 3.4%, OR, 1.979; 95% CI, 1.046–3.742; *P* = 0.033) than healthy controls (Table 2).

**Table 2:** Genotype and allele frequencies of rs148314165 and rs200820567 polymorphisms in patients with chronic HBV infection, HBV infection resolvers and healthy controls

Polymorphisms	Patients (*n* = 419)	Resolvers (*n* = 77)	Controls (*n* = 175)	*P*	Patients vs. resolvers		Patients vs. controls		Resolvers vs. controls	
					*P*	OR (95% CI)	*P*	OR (95% CI)	*P*	OR (95% CI)
rs148314165										
Genotype				0.053	0.190	1.803 (0.748–4.348)	0.028	2.069 (1.079–3.969)	0.791	0.871 (0.315–2.413)
TT	364 (86.9)	71 (92.2)	163 (93.1)							
Tdel	55 (13.1)	6 (7.8)	12 (6.9)							
Allele				0.063	0.205	1.733 (0.733–4.098)	0.033	1.979 (1.046–3.742)	0.794	1.142 (0.421–3.100)
T	783 (93.4)	148 (96.1)	338 (96.6)							
del	55 (6.6)	6 (3.9)	12 (3.4)							
rs200820567										
Genotype				0.053	0.190	1.803 (0.748–4.348)	0.028	2.069 (1.079–3.969)	0.791	0.871 (0.315–2.413)
TT	364 (86.9)	71 (92.2)	163 (93.1)							
TA	55 (13.1)	6 (7.8)	12 (6.9)							
Allele				0.063	0.205	1.733 (0.733–4.098)	0.033	1.979 (1.046–3.742)	0.794	1.142 (0.421–3.100)
T	783 (93.4)	148 (96.1)	338 (96.6)							
A	55 (6.6)	6 (3.9)	12 (3.4)							

Data are presented as n (%)

*HBV* hepatitis B virus, *OR* odds ratio, *95%CI* 95% confidence interval

### Genotype and allele frequencies of rs148314165, rs200820567 polymorphisms in hepatitis B virus (HBV) patients with different clinical diagnosis

The patients with chronic HBV infection included 165 chronic hepatitis, 158 liver cirrhosis, and 96 HCC in the clinical diagnosis. The genotype and allele frequencies of rs148314165, rs200820567 polymorphisms in HBV patients with different clinical diagnosis are shown in Table 3. There were no significant differences in the genotype and allele frequencies of rs148314165, rs200820567 polymorphisms between HBV patients with different clinical diagnosis (Table 3).

**Table 3:** Genotype and allele frequencies of rs148314165 and rs200820567 polymorphisms in patients with different clinical liver diseases of chronic HBV infection

Polymorphisms	Chronic hepatitis (*n* = 165)	Cirrhosis (*n* = 158)	HCC (*n* = 96)	*P*	Chronic hepatitis vs. Cirrhosis		Chronic hepatitis vs. HCC		Cirrhosis vs. HCC	
					*P*	OR (95% CI)	*P*	OR (95% CI)	*P*	OR (95% CI)
rs148314165										
Genotype				0.846	0.865	1.057 (0.559–1.997)	0.566	1.252 (0.581–2.695)	0.670	1.184 (0.544–2.579)
TT	142 (86.1)	137 (86.7)	85 (88.5)							
Tdel	23 (13.9)	21 (13.3)	11 (11.5)							
Allele				0.856	0.870	1.052 (0.570–1.942)	0.580	1.233 (0.587–2.588)	0.680	1.171 (0.552–2.486)
T	307 (93.0)	295 (93.4)	181 (94.3)							
del	23 (7.0)	21 (6.6)	11 (5.7)							
rs200820567										
Genotype				0.846	0.865	1.057 (0.559–1.997)	0.566	1.252 (0.581–2.695)	0.670	1.184 (0.544–2.579)
TT	142 (86.1)	137 (86.7)	85 (88.5)							
TA	23 (13.9)	21 (13.3)	11 (11.5)							
Allele				0.856	0.870	1.052 (0.570–1.942)	0.580	1.233 (0.587–2.588)	0.680	1.171 (0.552–2.486)
T	307 (93.0)	295 (93.4)	181 (94.3)							
A	23 (7.0)	21 (6.6)	11 (5.7)							

Data are presented as n (%)

*HBV* hepatitis B virus, *HCC* hepatocellular carcinoma, *OR* odds ratio, *95% CI* 95% confidence interval

The association of rs148314165 and rs200820567 polymorphisms with different clinical liver diseases of chronic HBV infection was further performed by logistic regression analysis with adjusting for covariates. The results showed that the rs148314165 and rs200820567 polymorphisms were not significantly associated with different clinical liver diseases of chronic HBV infection (Table 4).

**Table 4:** Association of rs148314165 and rs200820567 polymorphisms with different clinical liver diseases of chronic HBV infection by logistic regression analysis with adjusting for covariates

Variable	Chronic hepatitis vs. Cirrhosis			Chronic hepatitis vs. HCC			Cirrhosis vs. HCC			HCC vs. Patients without HCC		
	OR	SE	*P*	OR	SE	*P*	OR	SE	*P*	OR	SE	*P*
Gender	1.228	0.339	0.167	1.883	1.251	0.118	2.054	0.391	0.255	3.559	1.018	0.037
Age (years)	1.825	0.464	0.016	2.965	0.337	0.004	1.741	0.086	0.121	0.861	0.927	0.022
HBV DNA (IU/ml, log)	0.637	0.297	0.008	0.556	0.123	0.032	0.799	0.108	0.157	0.774	0.391	0.235
ALT (IU/L)	0.973	0.117	0.309	0.908	0.215	0.238	0.844	0.047	0.171	1.036	0.197	0.457
AST (IU/L)	0.965	0.106	0.413	0.927	0.333	0.165	0.893	0.085	0.106	1.072	0.105	0.371
Tbil (μmol/L)	0.833	0.225	0.274	1.101	0.079	0.067	1.099	0.027	0.367	0.907	0.043	0.556
Albumin (g/L)	0.276	0.362	0.096	0.861	0.122	0.104	0.875	0.102	0.225	1.113	0.068	0.173
*A20* rs148314165 polymorphism (T/T vs. T/del)	1.017	0.002	0.256	1.389	0.017	0.136	2.033	0.174	0.409	0.997	0.096	0.236
*A20* rs200820567polymorphism (T/T vs. T/A)	1.017	0.002	0.256	1.389	0.017	0.136	2.033	0.174	0.409	0.997	0.096	0.236

*HBV* hepatitis B virus, *HCC* hepatocellular carcinoma

### Haplotype frequencies of rs148314165, rs200820567 polymorphisms in hepatitis B virus (HBV) patients, HBV infection resolvers and healthy controls

Haplotypes of rs148314165, rs200820567 polymorphisms were constructed and compared in the study subjects. The assessment showed that the two polymorphisms (rs148314165 and rs200820567) are in complete linkage disequilibrium. Therefore, the haplotypes showed similar significance with the genotypes in the study subjects included. Namely, the haplotypes of rs148314165, rs200820567 polymorphisms between patients with chronic HBV infection and HBV infection resolvers, and between HBV infection resolvers and healthy controls had no significant differences, but the patients with chronic HBV infection had higher frequency of haplotypes delA + TA than healthy controls (6.6% vs. 3.4%; OR, 1.979; 95% CI, 1.046–3.742; *P* = 0.033, Additional file 1).

## Discussion

In view of the connection between A20 and HBV [11], HBV infection [12], liver inflammation [14], and HCC [16, 17], and the influence of *TNFAIP3* polymorphisms on A20 function and expression [18, 19, 22, 23] and liver regeneration [18], the *TNFAIP3* polymorphisms in patients with chronic HBV infection were investigated. In a previous study, the rs2230926 polymorphism, a nonsynonymous common coding SNP in exon 3 of *TNFAIP3,* which may alter A20 function [18], showed a lack of association with the susceptibility of chronic HBV infection or the progression of HBV-related diseases [21]. In the present study, the tandem polymorphisms (rs148314165, rs200820567), a pair of tandem polymorphic dinucleotides (deletion T followed by a T to A transversion), which were demonstrated to influence A20 expression through interfering with the delivery of NF-kB to the *TNFAIP3* promoter [22, 23], were examined in chronic HBV infection and HBV-related liver diseases.

The tandem polymorphisms (rs148314165, rs200820567) in *TNFAIP3* are collectively referred to as TT > A by virtue of the deletion T followed by a T to A transversion in the pair of rs148314165 and rs200820567 tandem polymorphic dinucleotides [22, 23]. PCR amplification followed by direct sequencing was used to determine the genotypes of the two polymorphisms (rs148314165 and rs200820567), respectively, in this study. The genotypes of rs148314165 and rs200820567 polymorphisms and the haplotypes constructed from the genotypes resulted in consistent findings. Namely, the genotype Tdel in rs148314165 is consistent with the genotype TA in rs200820567, and the allele del in rs148314165 is consistent with the allele A in rs200820567. Furthermore, the haplotypes delA + TA are also consistent with the allele del in rs148314165 or the allele A in rs200820567. These results confirm the successful determination of this TT > A polymorphic dinucleotide in *TNFAIP3*.

This study showed that the TT > A variant of the polymorphic dinucleotide (rs148314165,rs200820567) presented a significantly higher frequency in HBV patients than in healthy controls. The TT > A variant was also more frequent in HBV patients than in HBV infection resolvers although the difference was not significant. This TT > A variant was revealed to attenuate A20 expression through inefficient delivery of NF-kB to the *TNFAIP3* promoter [22, 23]. A20 was demonstrated to be a crucial hepatoprotective factor for chronic liver inflammation and cancer [14]. Consistently, the TT > A variant of the polymorphic dinucleotide (rs148314165,rs200820567) might contribute to the susceptibility of chronic HBV infection through attenuating A20 expression.

Although the *TNFAIP3* polymorphism rs2230926, which might influence the mRNA expression of *TNFAIP3* [19, 20], was not indicated to be associated with chronic HBV infection [21], the TT > A variant of the polymorphic dinucleotide (rs148314165,rs200820567), which may attenuate A20 expression, was indicated to be associated with the susceptibility of chronic HBV infection in this study. It is suggested that the TT > A variant of the polymorphic dinucleotide (rs148314165,rs200820567), in comparison with polymorphism rs2230926, might play a dominant effect on chronic HBV infection or the influences of the *TNFAIP3* polymorphism rs2230926 and the TT > A variant of the polymorphic dinucleotide (rs148314165,rs200820567) might be disease dependent. Definitely, further studies are needed to clarify the role of these two polymorphisms in human diseases including HBV infection and their possible differential predisposing effects on different diseases.

A20 has hepatoprotective effects because it could prevent chronic liver inflammation and reduce susceptibility to chemically or high fat-diet-induced HCC development in animals [14]. A20 could also promote liver regeneration [15] and inhibit HCC proliferation, metastasis and microvascular invasions [16, 17]. These functions of A20 in liver injury and HCC imply that *TNFAIP3* polymorphisms associated with the attenuation of A20 expression and/or the interference of A20 function, such as the TT > A variant investigated in this study, may be associated with disease progression and HCC development in chronic HBV infection. However, no significant association of the TT > A variant with different liver diseases including HCC in patients with chronic HBV infection was observed in this study. It should be pointed out that the sample size of patients including patients with HBV-related HCC is very small and this may contribute to the insignificance of the present study. Therefore, the potential link between *TNFAIP3* polymorphisms and the progression of liver diseases associated with chronic HBV infection needs further investigation in studies of larger sample size of patients.

Notably, A20 mRNA expression in peripheral blood mononuclear cells and its protein expression on monocytes were respectively indicated to be associated with the progression of chronic HBV infection and the severity of acute-on-chronic liver failure related to HBV infection [12, 13]. These findings are contradictory to the hepatoprotective and HCC inhibitory effects of A20 documented in other studies [14–17]. A possible explanation for this contradictory phenomenon of A20 may be the relative inadequaty in contrast to the severity of liver injury although the actual reasons and underlying mechanisms remain to be elucidated.

A20 may regulate NF-kB activity [4–6], and both A20 and NF-kB are complicated in HBV infection [8, 9, 11–13] and HBV-related [10] or non-HBV-related liver injury [14] and liver regeneration [15] as well as HCC proliferation and metastasis [16, 17]. A20 is involved in the regulation of the innate anti-viral immune response by targeting NF-kB and interferon regulatory factor pathways [26]. Considering these complex interactions between A20 and NF-kB, the involvement of A20 and NF-kB in HBV infection and the liver tissue homeostasis and the pathogenesis of liver diseases, and the role of A20 in the regulation of innate anti-viral immune response, the comprehensive link between *TNFAIP3* polymorphisms and chronic HBV infection and HBV-related diseases may be complex and needs in-depth studies.

## Conclusions

This study, for the first time to our knowledge, investigated the TT > A variant of the polymorphic dinucleotide (rs148314165,rs200820567) in *TNFAIP3* in Chinese patients with chronic HBV infection, and found that the TT > A variant was more frequent in patients with chronic HBV infection than in healthy controls, suggesting the predisposing effect of the TT > A variant on the susceptibility of chronic HBV infection. However, these findings were very preliminary because of the small sample size of patients and controls included in the study. The association analysis of the TT > A variant with clinical diseases were based on the crosssectional diseases of patients with chronic HBV infection. Therefore, additional studies in large patient and control populations, eapecially in patients with chronic HBV infection of longitudinal follow-up of the disease progression, are required to further clarify the role of this important TT > A variant of *TNFAIP3* in the susceptibility of chronic HBV infection and disease progression related to the infection.

## Additional file

Additional file 1: Haplotype frequencies of rs148314165, rs200820567 polymorphisms in patients with chronic HBV infection, HBV infection resolvers and healthy controls. (DOC 91 kb)
