# Supplementary material for: Association of the tandem polymorphisms (rs148314165, rs200820567) in TNFAIP3 with chronic hepatitis B virus infection in Chinese Han population
Source: Virol J. 2017 Aug 7;14:148. doi: 10.1186/s12985-017-0814-5 (PMC5547518; doi:10.1186/s12985-017-0814-5)
Supplement: Additional file 1: — Haplotype frequencies of rs148314165, rs200820567 polymorphisms in patients with chronic HBV infection, HBV infection resolvers and healthy controls. (DOC 91 kb) [file 12985_2017_814_MOESM1_ESM.doc]

Additional file 1

Haplotype frequencies of rs148314165, rs200820567 polymorphisms in patients with chronic HBV infection, HBV infection resolvers and healthy controls

| Haplotype | Patients (n = 419) | Resolvers (n = 77) | Controls (n = 175) | *P* | Patients *vs.* resolvers | | Patients *vs.* controls | | | Resolvers *vs.* controls | |
| --- | --- | --- | --- | --- | --- | --- | --- | --- | --- | --- | --- |
|  |  |  |  |  | *P* | OR (95% CI) | | *P* | OR (95% CI) | *P* | OR (95% CI) |
| TT | 783 (93.4) | 148 (96.1) | 338 (96.6) | Reference | Reference |  | | Reference |  | Reference |  |
| delA + TA | 55 (6.6) | 6 (3.9 ) | 12 (3.4) | 0.063 | 0.205 | 1.733 (0.733-4.098) | | 0.033 | 1.979 (1.046-3.742) | 0.794 | 1.142 (0.421-3.100) |

D’ = 1.000, r^2^ = 0.987; HBV, hepatitis B virus; OR, odds ratio; 95%CI, 95% confidence interval.
